# HMGA1 accelerates vascular smooth muscle cell osteogenic phenotype transdifferentiation by elevating pro-inflammatory cytokine IL-1β transcription in chronic kidney disease

**DOI:** 10.1038/s41420-026-03271-z

**Published:** 2026-08-04

**Authors:** Binhong Yang, Meijuan Cheng, Jingjing Jin, Kaixing Yan, Haoqi Sun, Xinyue Yang, Yunhui Wu, Qi An, Dongxue Zhang, Lixin Chang, Shenglei Zhang, Yaling Bai, Jinsheng Xu

**Affiliations:** 1 Department of Nephrology, The Fourth Hospital of Hebei Medical University, Shijiazhuang, China; 2 Hebei Clinical Research Center for Chronic Kidney Disease, Shijiazhuang, China; 3 Hebei Key Laboratory of Vascular Calcification in Kidney Disease, Shijiazhuang, China

**Keywords:** Genetics, Interleukins

## Abstract

Osteogenic differentiation of vascular smooth muscle cells (VSMCs) is recognized as a core pathological process underlying vascular calcification (VC). The inflammatory response contributes to the development of phenotypic switch, yet there is a lack of therapeutic strategies targeting this process. In the current study, we first identified that inflammatory factors are associated with the osteogenic differentiation of VSMCs, and Interleukin-1β (IL-1β) might be a key triggering mediator. Furthermore, elevated levels of IL-1β were observed in the calcified arteries of both VC patients and mice, and this increase was positively correlated with the severity of calcification. In vivo, treatment with the IL-1β pharmacological inhibition Canakinumab alleviated calcification and osteogenic conversion in the aortas of CKD mice. In vitro, overexpression of IL-1β exacerbated calcification and osteogenic differentiation by promoting inflammation in VSMCs, emphasizing the central role of pro-inflammatory factors. Mechanistically, HMGA1 was predicted to be a potential transcription factor of IL-1β. Specifically, HMGA1 directly promoted the expression of IL-1β by binding to its promoter region. Moreover, IL-1β inhibition nullified the pro-calcific effect of HMGA1 on VSMCs calcification and osteogenic differentiation, whereas IL-1β overexpression abolished the protective effect of HMGA1 silence. Finally, VSMC-specific HMGA1 deficiency in mice attenuated CKD-induced aorta calcification. Collectively, our findings reveal a novel transcriptional regulatory mechanism governing IL-1β expression, highlighting the HMGA1/IL-1β axis as a promising therapeutic target for VC in CKD.

## Introduction

Vascular calcification (VC), a pathological process characterized by ectopic hydroxyapatite deposition in blood vessel walls, is prevalent in individuals with atherosclerosis, chronic kidney disease (CKD), and diabetes mellitus [1,2]. In CKD patients, VC predominantly occurs in the vascular media, leading to vascular stiffness, increased pulse pressure, and ventricular hypertrophy, while also serving as a critical independent risk factor for adverse cardiovascular events [3]. Specifically, each 2.8-point increment in the coronary artery calcium score correlates with a significant 40% increase in cardiovascular risk [4]. Current therapeutic strategies for VC mainly target the risk factors driving its initiation and progression, including correcting calcium-phosphorus (Ca-Pi) metabolic disorders and modulating parathyroid hormone levels, yet effective interventions remain lacking. Thus, it is essential to elucidate the underlying molecular mechanisms of VC for optimizing clinical treatment.

The phenotypic transition of vascular smooth muscle cells (VSMCs) from a contractile phenotype to an osteogenic phenotype is a crucial pathological process in VC [5]. Persistent low-grade inflammation serves as a key driver for this process [6,7]. In CKD mouse models, inflammatory cytokines levels rise rapidly prior to VC, a consistent phenomenon also observed in high phosphate (Pi)-induced calcification of human VSMCs in vitro [8,9]. Cytokines mediate oxidative stress injury, apoptosis, and calcified vesicle release through their receptors and downstream signaling pathways, thereby promoting osteogenic differentiation of VSMCs [10]. The proinflammatory cytokine IL-1β downregulates bone morphogenetic protein type II receptor 2 (BMPR2) and activates the nuclear factor kappa B (NF-κB) signaling pathway, thereby increasing the expression of osteogenic transcription factors and bone matrix proteins [11,12]. Serum IL-18 levels positively correlate with coronary artery calcification and were found to activate the M-type transient receptor potential channel, thereby promoting high Pi-induced transdifferentiation and calcification in rat VSMCs [13]. Although significant progress has been made in elucidating the regulatory mechanisms of pro-inflammatory cytokines in VC in recent years, these findings have not yet been translated into effective therapeutic strategies. Therefore, further exploration of novel mechanisms to inhibit pro-inflammatory cytokine production is crucial.

High mobility group protein AT-hook1 (HMGA1) belongs to the non-histone chromosomal binding protein family [14]. It dynamically and reversibly binds to AT-rich minor grooves in the promoter regions of target genes, induces changes in chromatin conformation, and thus precisely modulates gene transcription [15]. It is involved in the regulation of various biological processes, including cell proliferation, chromatin remodeling, inflammation, and immune response [16–18]. A previous study demonstrated that under hypoxic conditions, HMGA1 upregulates COX-2 expression in vascular endothelial cells by binding to the AT-rich region near the NF-κB site within the COX-2 promoter [19]. In diabetic cardiomyopathy, overexpression of HMGA1 exacerbates high glucose-induced inflammatory responses in cardiomyocytes, leading to aggravated cardiac dysfunction [20]. Accumulating studies have revealed the regulatory role of HMGA1 in inflammation associated with cardiovascular diseases [21–23]. However, whether HMGA1 exerts its function by regulating inflammation in the context of VC remains to be explored.

In the present study, we investigated the biological function and upstream regulatory mechanism of the pro-inflammatory cytokine IL-1β in VC progression, and identified HMGA1 as a novel transcription factor for IL-1β. Specifically, HMGA1 directly transactivated the IL-1β gene by modulating its transcriptional activity, triggering a signaling cascade that eventually induced the upregulation of its downstream inflammation-related genes, thereby driving osteogenic differentiation and calcification of VSMCs in vitro and in vivo. Therefore, our study clarifies a novel transcriptional regulation mechanism of IL-1β and identifies the HMGA1/IL-1β/IL-6 axis as a potential therapeutic target for VC.

## Results

### Pro-inflammatory cytokines IL-1β and IL-6 are upregulated during VC

**Fig. 1:**
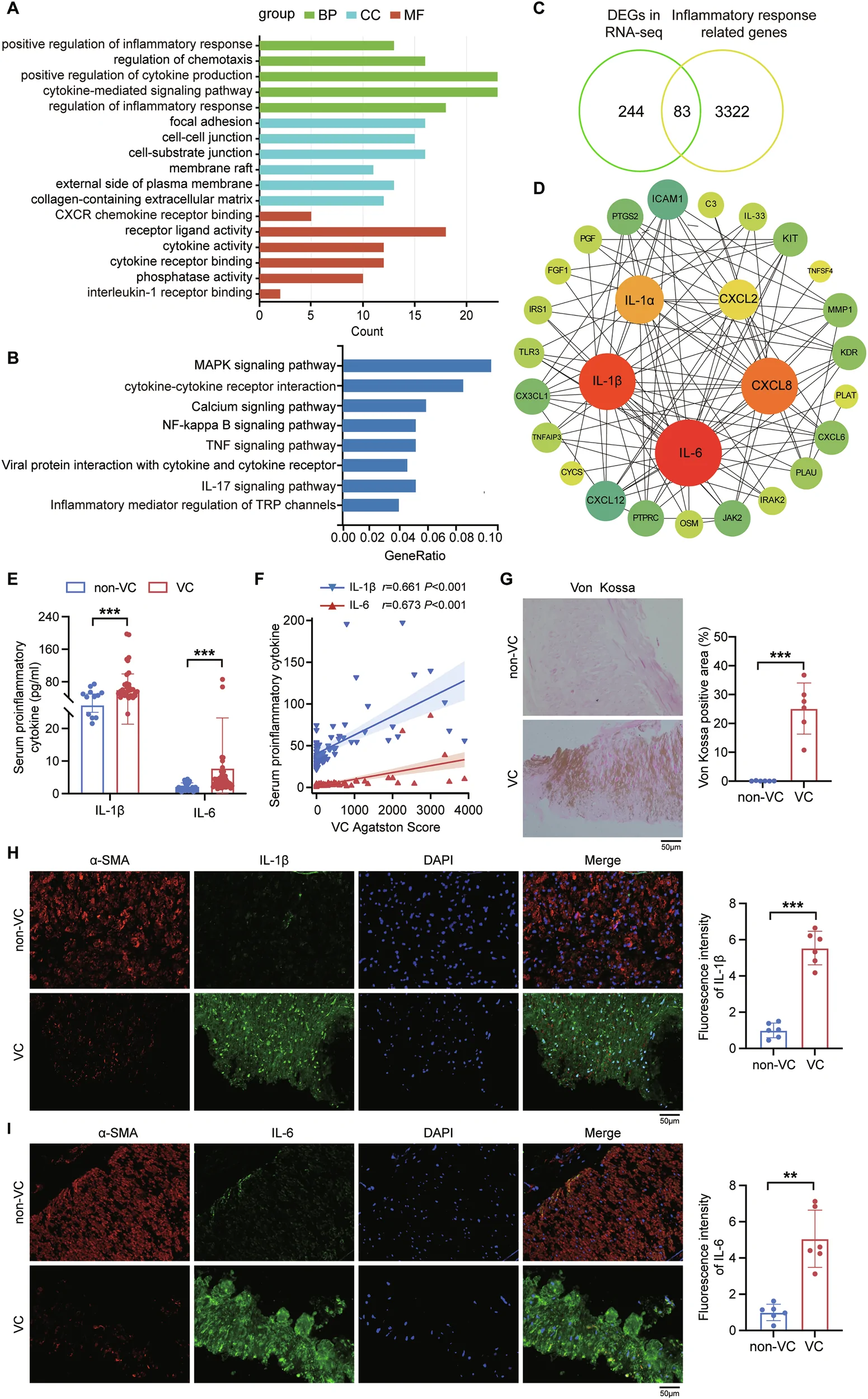
Pro-inflammatory cytokines IL-1β and IL-6 are upregulated during VC. **A**, **B** GO and KEGG enrichment analyses of DEGs in high Pi-induced human VSMCs. **C** Venn diagram showed the overlap between high Pi-induced DEGs in human VSMCs and inflammatory response- related genes. **D** PPI network of the inflammation-related DEGs. **E** Serum IL-1β and IL-6 between non-VC (*n* = 31) and VC (*n* = 49) groups of CKD patients. **F** Correlation analysis of serum IL-1β and IL-6 levels with the Agatston score in CKD patients (*n* = 31 for non-VC; *n* = 49 for VC). **G** Representative Von Kossa staining and quantitative analysis of radial arteries from non-VC and VC patients (*n* = 6 per group). Scale bars, 200 μm. **H** Representative immunofluorescence staining images of IL-1β and α-SMA in radial arteries from non-VC and VC groups (*n* = 6 per group). Scale bars, 50 μm. **I** Representative immunofluorescence staining images of IL-6 and α-SMA in radial arteries between non-VC and VC groups (*n* = 6 per group). Scale bars, 50 μm. Data are presented as mean ± SD. Correlation was determined via Spearman test. *P < 0.05, **P < 0.01, ***P < 0.001. VC vascular calcification, GO Gene Ontology, KEGG Kyoto Encyclopedia of Genes and Genomes, DEGs differentially expressed genes, CKD chronic kidney disease.

To reveal the potential pathogenic genes in VC, we performed microarray analysis on high Pi-induced human VSMCs and identified 327 differentially expressed genes (DEGs) using criteria of *P* < 0.05 and |log2 (fold change)| ≥ 1. GO analysis revealed significant enrichment of these genes in “positive regulation of cytokine production,” “cytokine-mediated signaling pathway,” “cytokine activity,” and “interleukin-1 receptor binding” (Fig. 1A). KEGG pathway analysis further demonstrated their significant enrichment in multiple inflammatory response-related pathways, including “cytokine-cytokine receptor interaction,” “NF-kappa B signaling pathway,” “TNF signaling,” and “IL-17 signaling pathway” (Fig. 1B). Hence, we intersected DEGs with the inflammatory response genes set (relevance score ≥6) from the GeneCards database, yielding 83 inflammation-related DEGs (Fig. 1C). Using the CytoHubba plugin in Cytoscape to identify the top hub genes revealed a close association between proinflammatory cytokines IL-6 and IL-1β and VC (Fig. 1D).

Subsequently, we found that serum IL-1β and IL-6 were significantly elevated in the calcified group of CKD patients compared with the non-calcified group (Fig. 1E). Further statistical analysis demonstrated positive correlation of both IL-1β (*r* = 0.661, *P* < 0.001) and IL-6 levels (*r* = 0.673, *P* < 0.001) with calcification score (Fig. 1F). After adjusting for age in a logistic regression, IL-1β and IL-6 were still independent risk factors for VC (Supplementary Table 5). To further validate their expression in the calcified artery, Von Kossa staining was performed to assess VC (Fig. 1G), and immunofluorescence (IF) staining was conducted to detect the expression of IL-1β and IL-6. Consistently, IL-1β and IL-6 expression was obviously increased in calcified arteries, with colocalization present with the VSMC marker α-SMA (Fig. 1H, I).

Building on these findings, we established a CKD-VC mouse model and found that the levels of IL-1β and IL-6 were also higher in the calcified aortic tissues than those in the control group (Supplementary Fig. 1A). Consistent with in vivo findings, both IL-1β and IL-6 mRNA were upregulated during high Pi-induced osteogenic differentiation of VSMCs (Supplementary Fig. 1B, C). Collectively, these findings indicated that pro-inflammatory cytokines IL-1β and IL-6 were closely associated with the development of VC.

### Inhibiting the pro-inflammatory cytokine IL-1β is a potential strategy to alleviate VC

**Fig. 2:**
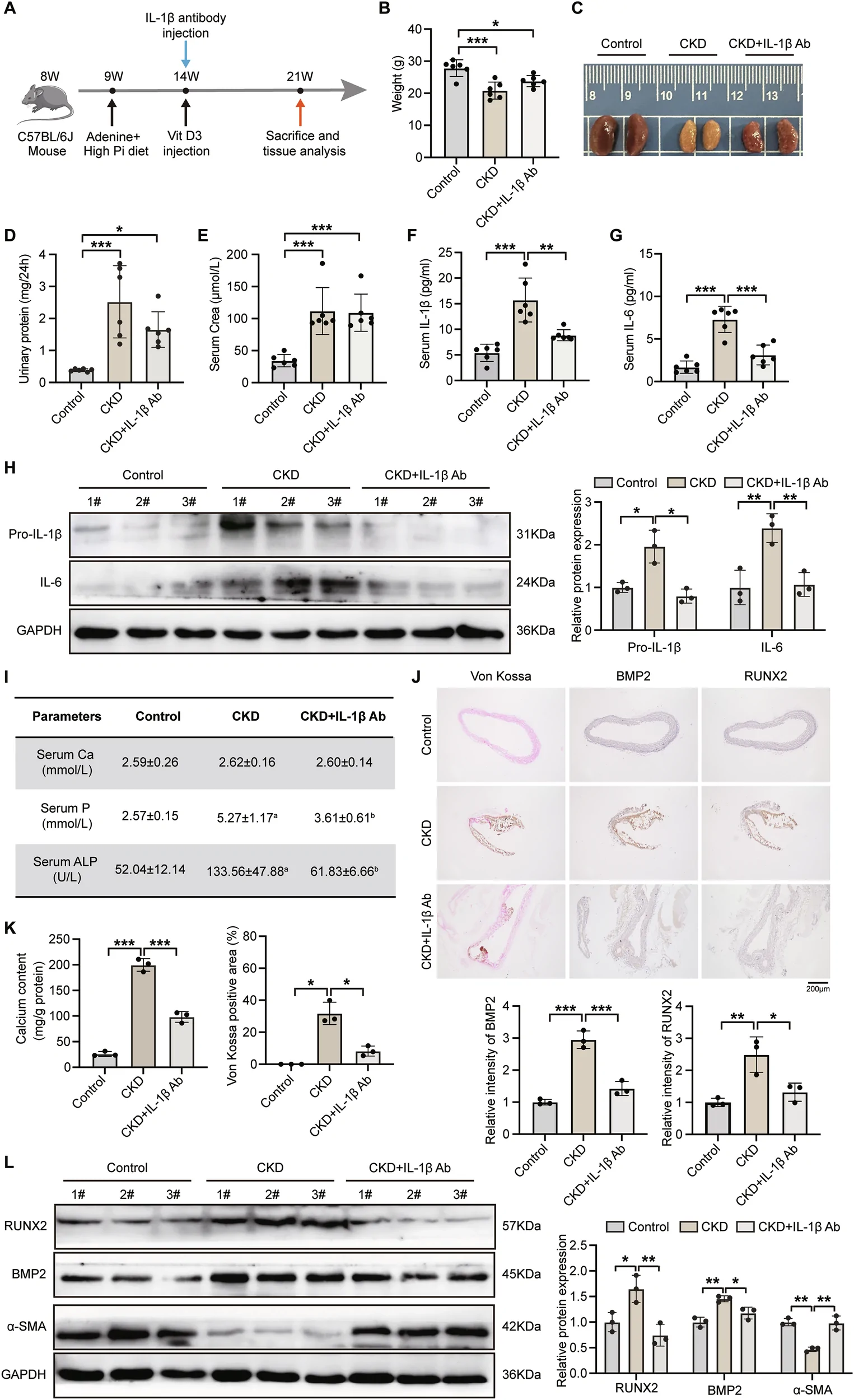
IL-1β inhibition alleviates VC in CKD mice. **A** Scheme of the treatment protocol with the IL-1β neutralizing antibody in the CKD mouse model. **B** Bodyweight (*n* = 6 per group). **C** Macroscopic observation of the kidney. **D** Urinary protein (*n* = 6 per group). **E** Serum creatinine (*n* = 6 per group) in control or CKD or CKD + IL-1β Ab mice. Serum levels of IL-1β (**F**) and IL-6 (**G**) in control or CKD or CKD + IL-1β Ab mice tested by ELISA kit (*n* = 6 per group). **H** Western blot analysis of Pro-IL-1β and IL-6 in aortic lysates from the indicated mouse groups (left), with quantitative data (right) (*n* = 3 per group). **I** Serum levels of Ca, Pi, and ALP in indicated mouse groups (*n* = 6 per group). a, means the difference between the control group; b, means the difference between the CKD group. **J** Representative Von Kossa and immunohistochemical staining for BMP2 and RUNX2 in aortic sections from different groups (*n* = 3 per group). Scale bars, 200 μm. **K** Calcium content quantification in the aortas of control, CKD or CKD + IL-1β Ab mice (*n* = 3 per group). **L** Western blot analysis of RUNX2, BMP2, and α-SMA in aortic lysates from the indicated mouse groups (left), with quantitative data (right) (*n* = 3 per group). Data are presented as mean ± SD. *P < 0.05, **P < 0.01, ***P < 0.001. VC vascular calcification, CKD chronic kidney disease, Ab antibody, Ca calcium, Pi phosphate, ALP alkaline phosphatase.

Given that IL-1β can initiate and amplify the inflammatory cascade by inducing the expression of various downstream inflammatory factors and chemokines during the inflammatory response [24,25], the IL-1β-neutralizing antibody canakinumab was used to assess its role in CKD mice with VC (Fig. 2A). The successful establishment of the CKD model was validated by hallmark features including reduced body weight, renal atrophy, irregular kidney surfaces, and elevated levels of urinary protein and serum creatinine (Fig. 2B–E). Next, we found that canakinumab treatment effectively suppressed IL-1β and consequently IL-6 levels in the serum (Fig. 2F, G), as well as in the aortic tissues (Fig. 2H). To investigate the impact of IL-1β inhibition on calcification, we subsequently evaluated key serological indicators and revealed that serum Pi levels and alkaline phosphatase (ALP) activity were significantly elevated in the CKD group compared with the control group, and these increases were mitigated after IL-1β inhibition (Fig. 2I). Furthermore, Von Kossa staining and calcium content analysis showed that IL-1β inhibition alleviated CKD-induced calcium deposits in mouse aortic tissues (Fig. 2J, K). Moreover, these observed effects were paralleled by attenuation of osteogenic change in the aortas, as inhibition of IL-1β could markedly enhance the expression of contractile marker α-SMA while decreasing the levels of osteogenic markers RUNX2 and BMP2 (Fig. 2J, L). In general, these findings suggested that inhibiting IL-1β could suppress aortic calcification through the modulation of osteogenic differentiation.

Based on the regulatory role of IL-1β in vivo, we then explored its effect on VSMCs cultured in vitro by transfecting the cells with an IL-1β-overexpressing plasmid. The transfection efficiency was verified at mRNA and protein levels (Supplementary Fig. 2A, B). The upregulation of IL-1β led to a significant increase in the protein level of IL-6 (Supplementary Fig. 2B). Of note, this upregulation of pro-inflammatory factors accelerated VSMCs calcification and osteogenic conversion (Supplementary Fig. 2C–E). In conclusion, these data provided evidence that the activation of inflammatory signaling exacerbated calcification formation by promoting osteogenic differentiation of VSMCs in vitro.

### HMGA1 is a novel transcription factor that regulates the inflammation in VC

**Fig. 3:**
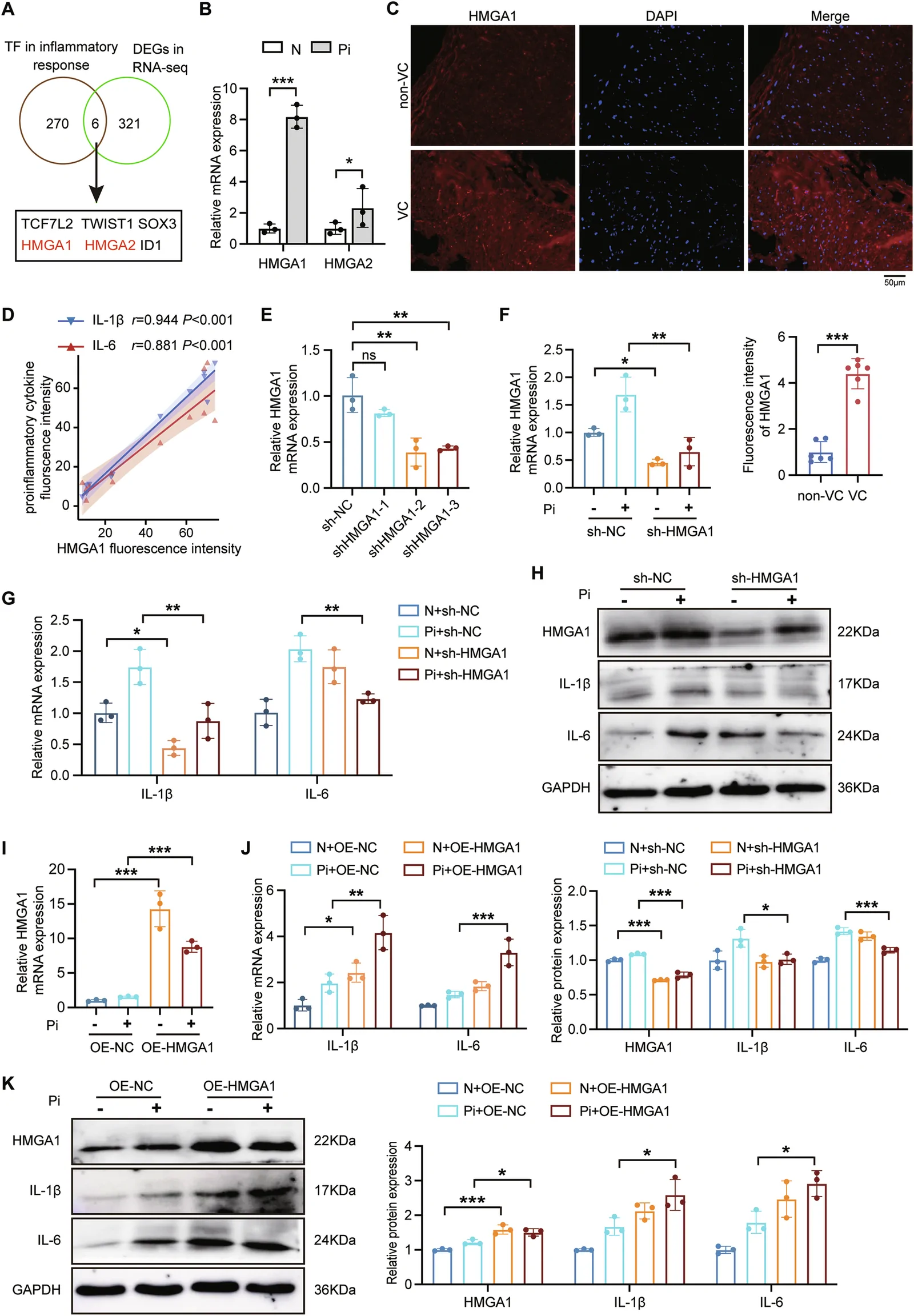
HMGA1 is a novel transcription factor that regulates the inflammatory response in VC. **A** Venn diagram showed the overlap between high Pi-induced DEGs in human VSMCs and transcription factors in inflammatory response. **B** The mRNA expression levels of HMGA1 and HMGA2 in VSMCs under Pi stimulation (*n* = 3 per group). **C** Immunofluorescence staining and quantification for HMGA1 in radial artery sections between non-VC and VC of CKD patients (*n* = 6 per group). Scale bars, 50 μm. **D** Correlation analysis between HMGA1, IL-1β and IL-6 immunofluorescence intensity in radial artery sections from CKD patients (*n* = 6 per group). **E** qRT-PCR showed transfection efficiency of sh-HMGA1-1 ~ 3 in Pi mediated VSMCs (*n* = 3). **F** Relative HMGA1 mRNA expression in N+sh-NC or Pi+sh-NC and N+sh-HMGA1 or Pi+sh-HMGA1 groups (*n* = 3). **G** Relative IL-1β and IL-6 mRNA expression in VSMCs after HMGA1 knockdown (*n* = 3). **H** Western blot analysis of HMGA1, IL-1β and IL-6 expression in VSMCs following HMGA1 knockdown (*n* = 3). **I** Relative HMGA1 mRNA expression in N + OE-NC or Pi+OE-NC and N + OE-HMGA1 or Pi+OE-HMGA1 groups (*n* = 3). **J** Relative mRNA expression of IL-1β and IL-6 in VSMCs after HMGA1 overexpression (*n* = 3). **K** Western blot analysis of HMGA1, IL-1β and IL-6 expression in VSMCs following HMGA1 overexpression (*n* = 3). Data are presented as mean ± SD. Correlation was determined via Spearman test. *P < 0.05, **P < 0.01, ***P < 0.001. VC vascular calcification, CKD chronic kidney disease, Pi phosphate, VSMCs vascular smooth muscle cells, N normal, sh-NC control shRNA, sh-HMGA1 shRNA for HMGA1, OE-NC control plasmid, OE-HMGA1 plasmid overexpressing HMGA1.

To investigate the potential transcriptional regulation network of inflammation in VC, we integrated inflammatory response-related transcriptional factors (TFs) with DEGs from RNA-seq, and identified six candidate genes (Fig. 3A). Of interest, both members of the HMGA protein family (HMGA1 and HMGA2) were aberrantly upregulated under this condition. Hence, we examined their mRNA levels and found that HMGA1 expression was significantly higher than that of HMGA2 in calcified VSMCs, prompting us to select HMGA1 for further investigation (Fig. 3B). Subsequently, we observed an increase in the protein level of HMGA1 in calcified artery specimens, as evidenced by the enhanced intensity of red fluorescence (Fig. 3C). Further correlation analysis of the immunofluorescence staining of arterial tissues from patients indicated a strong positive correlation of HMGA1 with both IL-1β (r = 0.944, *P* < 0.001) and IL-6 (r = 0.755, *P* = 0.006) (Fig. 3D). To determine whether HMGA1 regulated vascular inflammation, VSMCs were transfected with three specific short hairpin RNAs targeting HMGA1. Among them, sh-HMGA1-2 was chosen for subsequent experiments based on its superior knockdown efficiency (Fig. 3E). As expected, HMGA1 was significantly downregulated in both the normal and Pi medium compared with the corresponding control group (Fig. 3F, H). HMGA1 knockdown induced an obvious decrease in IL-1β and IL-6 expression, both at the mRNA and protein levels, in VSMCs cultured in a calcified medium (Fig. 3G, H). Conversely, overexpression of HMGA1 significantly increased the expression of inflammatory cytokines in VSMCs under calcified conditions (Fig. 3I, K). Taken together, these findings demonstrated that HMGA1 could serve as a crucial transcriptional regulator driving the expression of pro-inflammatory cytokines during VC.

### IL-1β is transcriptionally promoted by HMGA1

**Fig. 4:**
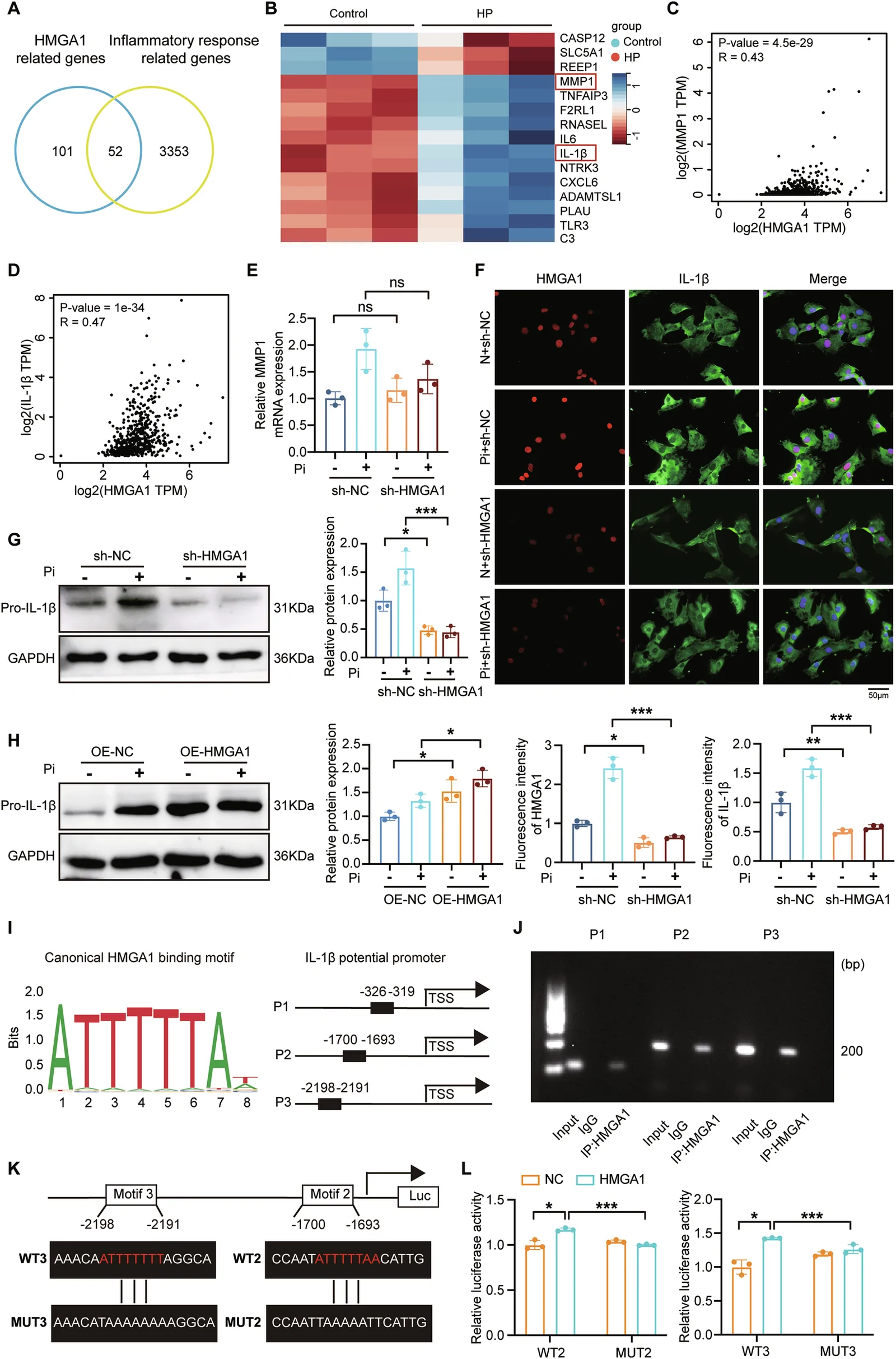
IL-1β is transcriptionally promoted by HMGA1. **A** Venn diagram showed the overlap between HMGA1-related genes and inflammatory response related genes. **B** Heatmap of the top 15 genes co-expressed with HMGA1 based on RNA-seq data from VSMCs treated with high Pi or normal medium. The correlation of HMGA1 and MMP1 (**C**) or IL-1β (**D**) in human aorta from the GTEx database. Spearman analysis was performed using the webtool GEPIA. **E** qRT-PCR of MMP1 expression in VSMCs after HMGA1 knockdown (*n* = 3). **F** The colocalization of IL-1β (green) and HMGA1 (red) in VSMCs was detected by double-labeling immunofluorescence (*n* = 3). Scale bars, 50 μm. **G** Western blot analysis of Pro-IL-1β expression in VSMCs following HMGA1 knockdown (*n* = 3). **H** Western blot analysis of Pro-IL-1β expression in VSMCs following HMGA1 overexpression (*n* = 3). **I** The binding site sequence of HMGA1 was acquired from the JASPAR database. **J** CHIP-PCR assay confirmed that the DNA fragment corresponding to the promoter of IL-1β enriched by HMGA1 binding. **K**, **L** Relative luciferase activity of HEK293T cells transfected with WT and Mut reporter constructs along with HMGA1 overexpression or empty vector (NC). *n* = 3. Data are presented as mean ± SD. Correlation was determined via Spearman test. * *P* < 0.05, ** *P* < 0.01, *** *P* < 0.001, ns not statistically significant. VC vascular calcification, VSMCs vascular smooth muscle cells, Pi phosphate, CHIP chromatin immunoprecipitation.

To uncover the underlying molecular mechanism by which HMGA1 exacerbated vascular inflammation, we leveraged the microarray dataset from VSMCs treated with high Pi to identify HMGA1 co-expressed genes, and performed an intersection analysis with inflammatory response-related genes (Fig. 4A). Supplementary Table 6 lists the top 15 HMGA1 co-expressed genes ranked by correlation coefficient. Heatmap analysis revealed MMP1 and IL-1β as the most significantly altered candidate downstream targets among these genes (Fig. 4B). Next, using the GTEx database, we found that HMGA1 was more highly correlated with IL-1β than with MMP1 in the human aorta (Fig. 4C, D). Similarly, silencing HMGA1 reduced IL-1β mRNA under both normal and Pi conditions (Fig. 3G), whereas MMP1 expression was unaffected (Fig. 4E). These changes were confirmed at the protein level by immunofluorescence staining, which revealed that HMGA1 was significantly decreased in HMGA1 knockdown group along with a corresponding reduction in IL-1β compared with controls (Fig. 4F). Western blot assay corroborated these findings, demonstrating a clear reduction in pro-IL-1β protein (Fig. 4G). Conversely, overexpression of HMGA1 increased IL-1β both at the mRNA and protein levels compared with the corresponding group (Figs. 3J and 4G).

Since HMGA1 is a chromatin structure modifier and our data indicated it positively regulated IL-1β, we hypothesized that it might modulate IL-1β expression at the transcriptional level. Preliminarily, the JASPAR database was utilized to identify the binding site sequence of HMGA1 and predict three potential binding sites (labeled P1, P2 and P3) within the IL-1β promoter region at specific locations from the transcription start site (Fig. 4I). Based on the predicted results, a ChIP-PCR assay was conducted and the enrichment of HMGA1 detected within P2 (−1700 to −1693 bp) and P3 (−2198 to −2191 bp) sites was markedly stronger than that detected within P1 site (Fig. 4J). Next, luciferase reporter vectors containing wild-type (WT) or mutant (Mut) of P2 and P3 sites were constructed and co-transfected with an HMGA1 overexpression plasmid into HEK293T cells to examine the luciferase activity. Notably, HMGA1 overexpression activated the relative luciferase activity of the reporter vector containing the WT IL-1β promoter, and this effect was abolished after transfection with the Mut reporter genes (Fig. 4K, L). In conclusion, our findings demonstrated that HMGA1 enhanced the transcriptional level of IL-1β by directly binding to its promoter.

### IL-1β inhibition reverses the pro-calcific effect of HMGA1

**Fig. 5:**
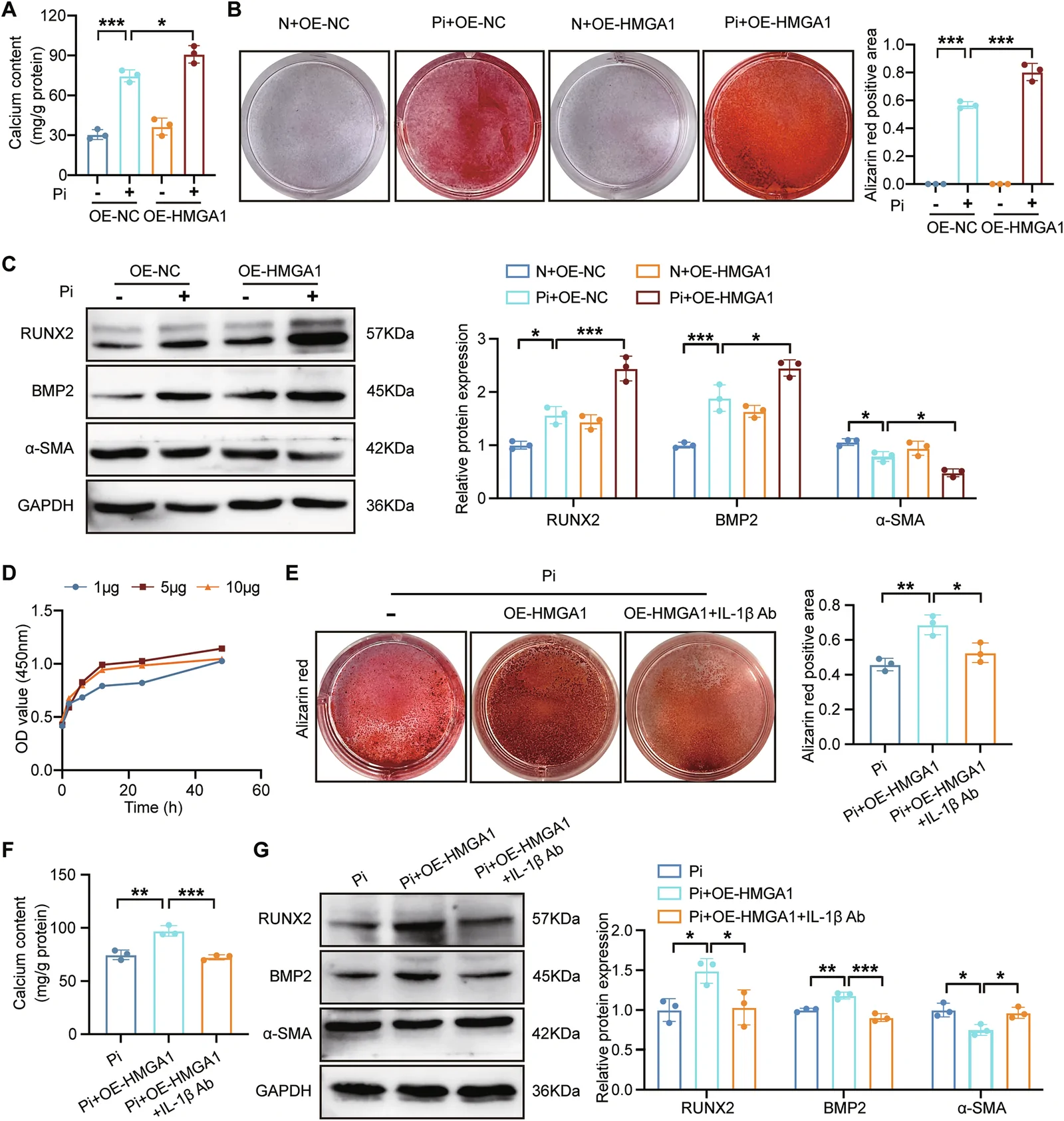
IL-1β inhibition reverses the pro-calcific effect of HMGA1. **A** Calcium content quantification of VSMCs in N + OE-NC or Pi+OE-NC and N + OE-HMGA1 or Pi+OE-HMGA1 groups (*n* = 3). **B** Representative images and quantification of alizarin red staining of VSMCs (*n* = 3). **C** Western blot analysis of RUNX2, BMP2 and α-SMA of VSMCs under various transfection together with or without Pi stimulus. **D** Cell viability was measured in VSMCs exposed to Pi medium and treated with different IL-1β antibody concentrations for the indicated times (*n* = 3). **E** Representative images and quantification of alizarin red staining for different pretreatments (*n* = 3). **F** Quantification of calcium content in VSMCs under Pi stimulus together with HMGA1 overexpression plasmid (OE-HMGA1) or IL-1β antibody for 48 h (*n* = 3). **G** Western blot analysis of RUNX2, BMP2, and α-SMA with Pi stimulus together with indicated transfection or IL-1β antibody (*n* = 3). Data are presented as mean ± SD. *P < 0.05, **P < 0.01, ***P < 0.001. Pi phosphate, VSMCs vascular smooth muscle cells, N normal, OE-NC control plasmid, OE-HMGA1 plasmid overexpressing HMGA1.

Considering that HMGA1 was upregulated in VC and activated the pro-calcific factor IL-1β expression, we aimed to investigate the potential function of HMGA1 in this process. As expected, overexpression of HMGA1 markedly promoted the formation of intracellular calcium nodules and the mineralization process within VSMCs compared with the control group (Fig. 5A, B). Consistently, elevated HMGA1 upregulated osteogenic markers and downregulated contractile marker (Fig. 5C). To validate that IL-1β is required for HMGA1-mediated calcification, VSMCs overexpressing HMGA1 were treated with the IL-1β neutralizing antibody canakinumab. We first evaluated the potential cytotoxicity of the IL-1β neutralizing antibody canakinumab on VSMCs. CCK-8 assays showed no significant cytotoxicity of canakinumab within the concentration range of 1–10 μg/mL, and 5 μg/mL was therefore selected for subsequent experiments (Fig. 5D). Expectedly, the inhibition of IL-1β partially reversed HMGA1-induced VSMC calcification, as demonstrated by the decreased calcium deposition (Fig. 5E). Likewise, the elevation in the intensity of alizarin red staining induced by HMGA1 overexpression was effectively abolished by IL-1β inhibition (Fig. 5F). Furthermore, IL-1β suppression prevented HMGA1-mediated upregulation of RUNX2 and BMP2 and downregulation of α-SMA (Fig. 5G). In conclusion, the above data demonstrated that the pro-calcific role of HMGA1 is partially dependent on IL-1β.

### HMGA1 deficiency exerts a protective effect on VSMCs calcification through IL-1β

**Fig. 6:**
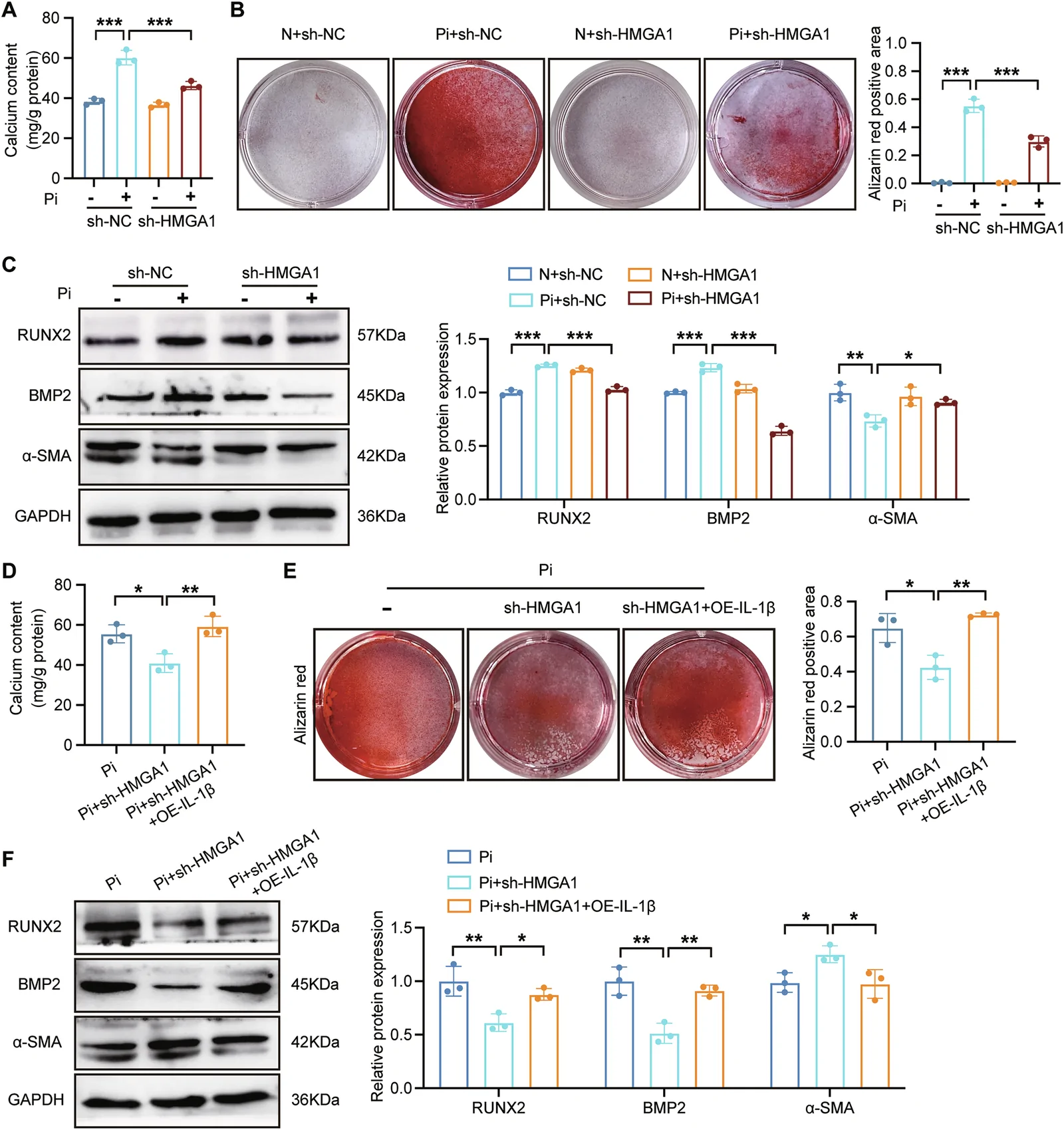
HMGA1 deficiency exerts a protective effect on VSMCs calcification through IL-1β. **A** Calcium content quantification of VSMCs in N+sh-NC or Pi+sh-NC and N+sh-HMGA1 or Pi+sh-HMGA1 groups (*n* = 3). **B** Alizarin red staining and quantification in VSMCs under the indicated treatments (*n* = 3). **C** Western blot analysis of RUNX2, BMP2 and α-SMA of VSMCs under various transfection together with or without Pi stimulus. **D** Quantification of calcium content in VSMCs transfected with sh-HMGA1, followed by transfection with OE-IL-1β under Pi stimulus (*n* = 3). **E** Representative alizarin red staining and quantification of in VSMCs transfected with sh-HMGA1, followed by transfection with OE-IL-1β under Pi stimulus (*n* = 3). **F** Western blot analysis and quantification of RUNX2, BMP2, and α-SMA in VSMCs with indicated transfection (*n* = 3). Data are presented as mean ± SD. *P < 0.05, **P < 0.01, ***P < 0.001. Pi phosphate, VSMCs vascular smooth muscle cells, N normal, sh-NC control shRNA, sh-HMGA1 shRNA for HMGA1, OE-IL-1β plasmid overexpressing IL-1β.

To further determine the role of HMGA1 in VC, we investigated whether HMGA1 knockdown suppresses the transformation of VSMCs into the osteogenic phenotype and impedes calcification. The sh-HMGA1-2 was transfected into VSMCs, and HMGA1 knockdown mitigated Pi-induced calcium deposition in VSMCs (Fig. 6A). The impact of HMGA1 on VSMC calcification was further verified by the reduced formation of calcified nodules, as observed by alizarin red staining (Fig. 6B). Moreover, HMGA1 deficiency negated the increase in the osteogenic protein expression, including BMP2 and RUNX2, caused by Pi medium and increased the expression of α-SMA, suggesting that HMGA1 knockdown suppressed osteogenic phenotypic differentiation of VSMCs (Fig. 6C). Therefore, we focused on exploring whether HMGA1 inhibited VSMCs osteogenic transdifferentiation and calcification in an IL-1β-dependent manner. Interestingly, the protective effects of HMGA1 deficiency in the mitigation of calcification were partly abolished in the presence of IL-1β overexpression, as evidenced by changes in quantification of calcium content and alizarin red staining (Fig. 6D, E). Simultaneously, overexpression of IL-1β significantly aggravated osteogenic transdifferentiation under the condition of HMGA1 knockdown (Fig. 6F). Altogether, these results supported the conclusion that HMGA1 alleviated VSMCs osteogenic transdifferentiation and calcification via IL-1β.

### HMGA1 ameliorates arterial inflammation and calcification in mice with CKD

**Fig. 7:**
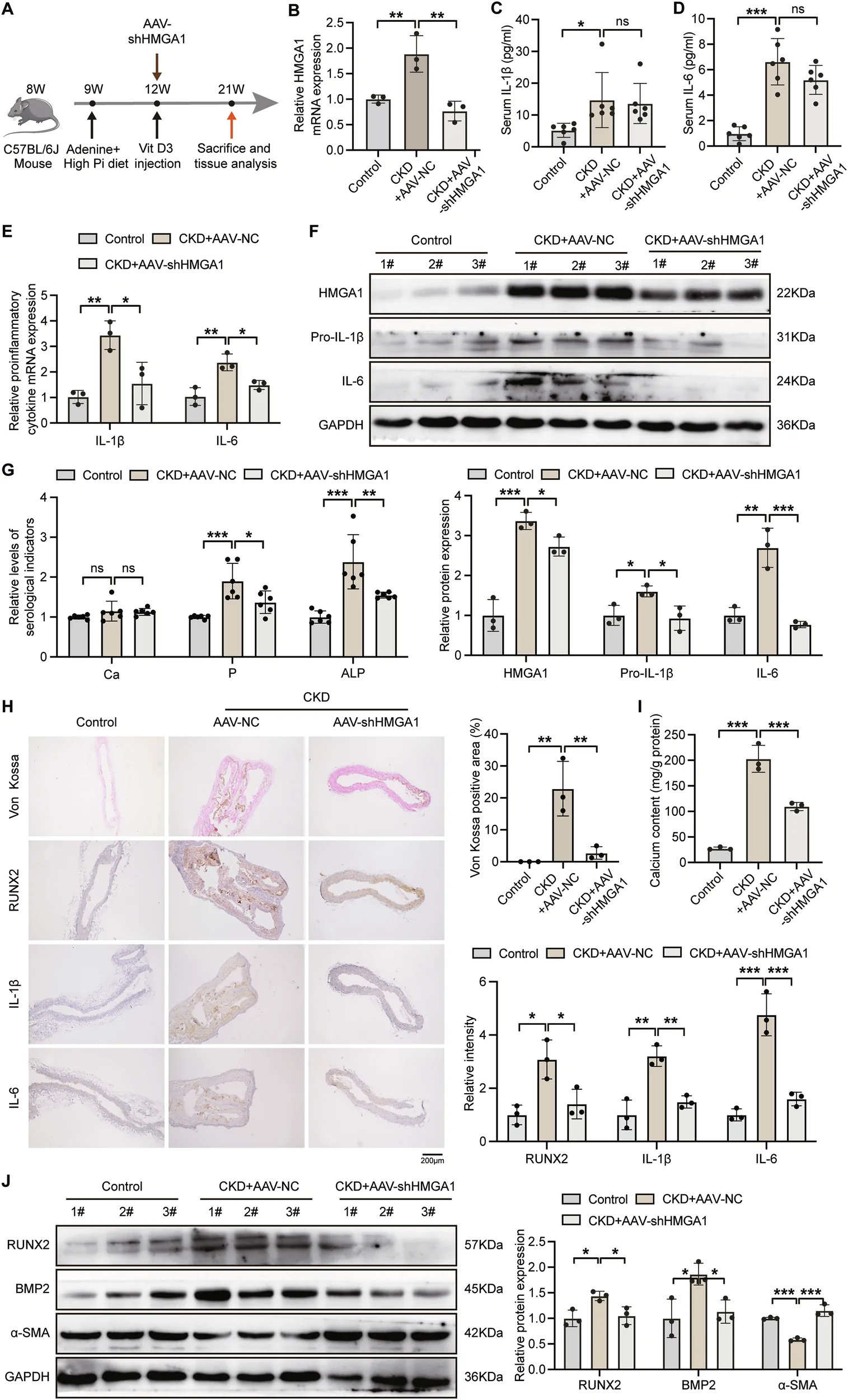
HMGA1 knockdown ameliorates inflammation and arterial calcification in mice with CKD. **A** Schematic of tail vein injection of AAV-NC or AAV-shHMGA1 in CKD mice. **B** HMGA1 knockdown in arteries confirmed by qRT-PCR. *n* = 3 per group. Serum levels of IL-1β (**C**) and IL-6 (**D**) in control or CKD + AAV-NC or CKD + AAV-shHMGA1 mice tested by ELISA kit. *n* = 6 per group. **E** The mRNA levels of IL-1β and IL-6 in aortic tissues from experimental groups. *n* = 3 per group. **F** Western blot and quantification analysis of HMGA1, Pro-IL-1β and IL-6 in aortic tissues from experimental groups. *n* = 3 per group. **G** Serum levels of Ca, Pi, and ALP in indicated mouse groups. *n* = 6 per group. **H** Von Kossa and immunohistochemical staining of RUNX2, IL-1β and IL-6 in aortic sections of CKD mice with indicated treatment. *n* = 3 per group. Scale bars, 200 μm. **I** Calcium content quantification of aortas normalized to protein content. *n* = 3 per group. **J** Western blot analysis of osteogenic phenotypic markers including RUNX2, BMP2 and contractile marker α-SMA in aortic tissues among all groups. *n* = 3 per group. Data are presented as mean ± SD. *P < 0.05, **P < 0.01, ***P < 0.001, ns not statistically significant. VSMCs vascular smooth muscle cells, CKD chronic kidney disease, Ca calcium, Pi phosphate, ALP alkaline phosphatase, AAV adeno-associated virus.

To further evaluate the role of HMGA1 in VC in vivo, we delivered AAV9 vectors carrying either scrambled shRNA (AAV-NC) or HMGA1 shRNA (AAV-shHMGA1) to manipulate HMGA1 expression in CKD mice (Fig. 7A). CKD mice exhibited obvious renal failure, confirmed by reduced body weight and kidney size, along with increased urinary protein and serum creatinine, yet there were no significant differences between the AAV-NC and AAV-shHMGA1 groups (Supplementary Fig. 3A–D). HMGA1 was successfully knocked down in the aorta of mice (Fig. 7B, F), leading to a reduction in the expression of IL-1β and IL-6 in aortic tissues without influencing their serum concentrations, which suggested that HMGA1 likely functions primarily by modulating the local inflammatory microenvironment within the vasculature during calcification (Fig. 7C–F). This protein level reduction of inflammation cytokines was further confirmed by the lower intensity of IHC in AAV-shHMGA1 mice than in AAV-NC mice (Fig. 7H). Subsequently, we assessed the calcification status of the mice and found that HMGA1 inactivation reduced serum levels of Pi and ALP, which were closely linked to the occurrence of VC (Fig. 7G). In addition, reduced mineral deposition was detected in HMGA1 knockdown mice compared to the scramble mice under CKD conditions, accompanied by a decreased level of osteochondrogenic marker RUNX2 (Fig. 7H, I). In line with previous observation, Western blot further demonstrated that HMGA1 knockdown dampened osteogenic phenotypic transdifferentiation (Fig. 7J). Collectively, our results indicated that reduced HMGA1 expression blunts vascular inflammation and calcification in vivo.

## Discussion

VC has been established as a serious threat to both cardiovascular health and survival in patients with CKD [26,27]. This process involves intricate mechanisms, and no effective treatment is currently available. Therefore, elucidating the pathogenesis of VC and identifying viable therapeutic targets are clinically significant [28]. Our study revealed the pro-inflammatory factors IL-1β and IL-6 as major contributors to VSMC osteogenic differentiation, as evidenced by our integrated in vivo and in vitro findings that exogenous overexpression of IL-1β exacerbated Pi-induced VC progression, while anti-IL-1β therapy exerted a distinct anti-calcific effect. Further mechanistic investigation revealed that the transcription factor HMGA1 acts as a key upstream regulator of IL-1β, directly binding to specific sites on the IL-1β promoter to regulate its transcription. In addition, we found that the inhibition of IL-1β counteracted the pro-calcific impact of HMGA1 on VSMCs calcification and osteogenic differentiation. Conversely, the overexpression of IL-1β negated the protective effect of HMGA1 silencing. Finally, VSMC-specific HMGA1 deficiency in mice attenuated CKD-induced aortic calcification. Collectively, the HMGA1–IL-1β–IL-6 signaling axis plays a central role in VC, suggesting that co-targeting this axis may offer further translational potential for its clinical treatment.

IL-1β, as a pivotal pro-inflammatory cytokine, is involved in the progression of various diseases including cancer, cardiovascular disorders, and numerous autoimmune and inflammatory conditions [29–31]. The pro-atherogenic role of IL-1β has been fully documented. Patients with atherosclerosis exhibited significantly elevated levels of IL-1β protein and mRNA, which contributed to the entire spectrum of disease progression, from the early initiation of inflammation to late-stage thrombotic complications [31,32]. Similarly, we observed significantly elevated serum levels of IL-1β and its downstream inflammatory cytokine IL-6 in patients with calcific CKD, the levels of which positively correlated with the severity of VC. Consistent with human data, arterial tissues from a CKD mouse model also showed increased levels of pro-inflammatory factors. A recent study demonstrated that circulating IL-1β has predictive value for the prognosis of acute decompensated heart failure [33]. Our results might propel the development of IL-1β as a potential biomarker to diagnose VC in patients with CKD.

Given the crucial role of IL-1β in inflammatory activation, several IL-1β-targeting agents have shown therapeutic potential across multiple diseases. For instance, IL-1β monoclonal antibodies have been approved for the treatment of rheumatic diseases and have demonstrated clinical benefits in reducing inflammation-driven atherosclerosis risk in the CANTOS trial [24,34]. Additionally, Awan et al. found that the anti-IL-1β monoclonal antibody attenuates VC in a mouse model of familial hypercholesterolemia [35]. Our study demonstrated that IL-1β monoclonal antibody treatment exerted potent anti-VC effects in a CKD mouse model. Our preclinical evidence aligns with clinical observations in a CKD cohort with myocardial infarction, in which canakinumab markedly lowered adverse cardiovascular events, particularly among patients with a marked initial anti-inflammatory response, and all without compromising renal function [36]. This renal safety is a pivotal factor in managing CKD patients, especially those in non-end stage kidney disease. However, studies in ApoE^−/−^ mouse models did not show a protective effect from IL-1β inhibition [37]. This discrepancy reveals differences in disease pathogenesis between that driven by pure hyperlipidemia and that driven by the uremic-inflammatory milieu of CKD-VC. Consequently, the efficacy of anti-IL-1β therapy is likely contingent upon specific metabolic and inflammatory backgrounds. Further exploration is needed to elucidate the relationship between IL-1β antibody and CKD-VC and to identify the patient subpopulations most likely to benefit from this targeted strategy.

Canonically, IL-1β activation is induced by the transcription factor (NF-κB) downstream of pattern recognition receptors [38]. However, growing evidence suggests the existence of alternative transcriptional regulatory mechanisms that are independent of the classical inflammasome pathway. For instance, in thoracic aortic aneurysm, loss of Sirt6 upregulates the transcription factor IRF8, thereby promoting IL-1β gene transcription [39]. Additionally, IL-1β is transcriptionally regulated by STAT4 in BaP/BPDE-induced miscarriage [40]. In contrast to transcriptional activation, counter-regulatory mechanisms exist. Nuclear factor erythroid 2-related factor 2 (Nrf2) has been shown to suppress inflammatory responses in macrophages by inhibiting transcriptional initiation at the promoter regions of IL-6 and IL-1β genes, as well as the recruitment of RNA polymerase II, thereby preventing their transcription [41]. This accumulating evidence indicates that precise regulation of IL-1β in different tissues and diseases requires specific transcriptional programs. To our knowledge, our study is the first to identify HMGA1 as a direct transcriptional regulator of IL-1β expression in VC, thereby enriching the molecular regulatory network of IL-1β-mediated vascular pathological remodeling. We demonstrated that loss of HMGA1 in both in vivo and in vitro models of vascular smooth muscle cells led to significant reductions in IL-1β mRNA and protein expression, whereas HMGA1 overexpression resulted in their upregulation. These findings not only offer new insights into the role of IL-1β in chronic diseases but also suggest that targeting its transcriptional regulation may represent a novel strategy for suppressing the pathological activity of IL-1β. We did not assess the expression or activation status of inflammasome components in our experimental models. Therefore, it remains to be determined whether the observed HMGA1-mediated upregulation of IL-1β occurs independently of or in concert with the canonical inflammasome pathway. Further investigations are warranted to clarify the potential crosstalk between HMGA1-driven transcriptional regulation and inflammasome-mediated inflammatory signaling in VC.

Although the oncogenic role of HMGA1 has received extensive attention from many researchers [42,43], little is known about its role in vascular function and calcification. In this study, we observed significant upregulation of HMGA1 expression in Pi-induced VSMCs, a CKD mouse model, and human calcified arterial tissues, suggesting its potential involvement in the pathogenesis of VC. Through gain- and loss-of-function experiments, we further confirmed the critical role of HMGA1 in regulating the osteogenic differentiation of VSMCs. In vivo knockdown of HMGA1 effectively ameliorated arterial calcification in mice with CKD. As an architectural transcription factor, HMGA1 modulates gene transcription. Typically, highly expressed during embryonic development but maintained at low levels in normal adult tissues, HMGA1 appears to be important in regulating proliferation and differentiation. For example, Song et al. identified HMGA1 as a novel transcription factor for α-SMA during mouse dorsal skin wound healing, which reduces fibroblast differentiation while promoting migration [44]. Similarly, Xie et al. reported that HMGA1 overexpression activates fibroblasts by increasing FOXO1 transcription, thereby promoting cardiac fibrosis [45]. Consistent with its broader regulatory functions, our findings reveal that elevated HMGA1 promotes IL-1β expression in VSMCs, facilitating their transition to an osteoblast-like phenotype. This aligns with its established role in skeletal biology, where HMGA1 regulates osteogenic differentiation of bone marrow mesenchymal stem cells via the Wnt/β-catenin signaling pathway [46]. Given the mechanistic similarities between vascular calcification and bone mineralization, HMGA1 emerges as a promising therapeutic target for VC.

In fact, this study did not employ specific small-molecule inhibitors targeting HMGA1 for validation. Our genetic loss-of-function approaches definitively established its critical role in VC. Of note, a recent study on the therapeutic potential of HMGA1 reported a novel nanoscale inhibitor, PDIC-DPC. This compound exhibits potent HMGA1-inhibitory activity by forming hydrogen bonds with Arg45 and Gly48 residues. In models of lung metastasis and orthotopic esophageal squamous cell carcinoma, PDIC-DPC treatment effectively downregulated HMGA1 expression without inducing systemic toxicity [47]. The ability of this nanoparticle to counteract HMGA1-mediated immunosuppression presents a promising therapeutic strategy. These findings suggest that targeting HMGA1 may hold potential therapeutic value for intervening in VC. Future studies should further explore the precise role of HMGA1 in VC and its translational potential.

## Conclusion

**Fig. 8:**
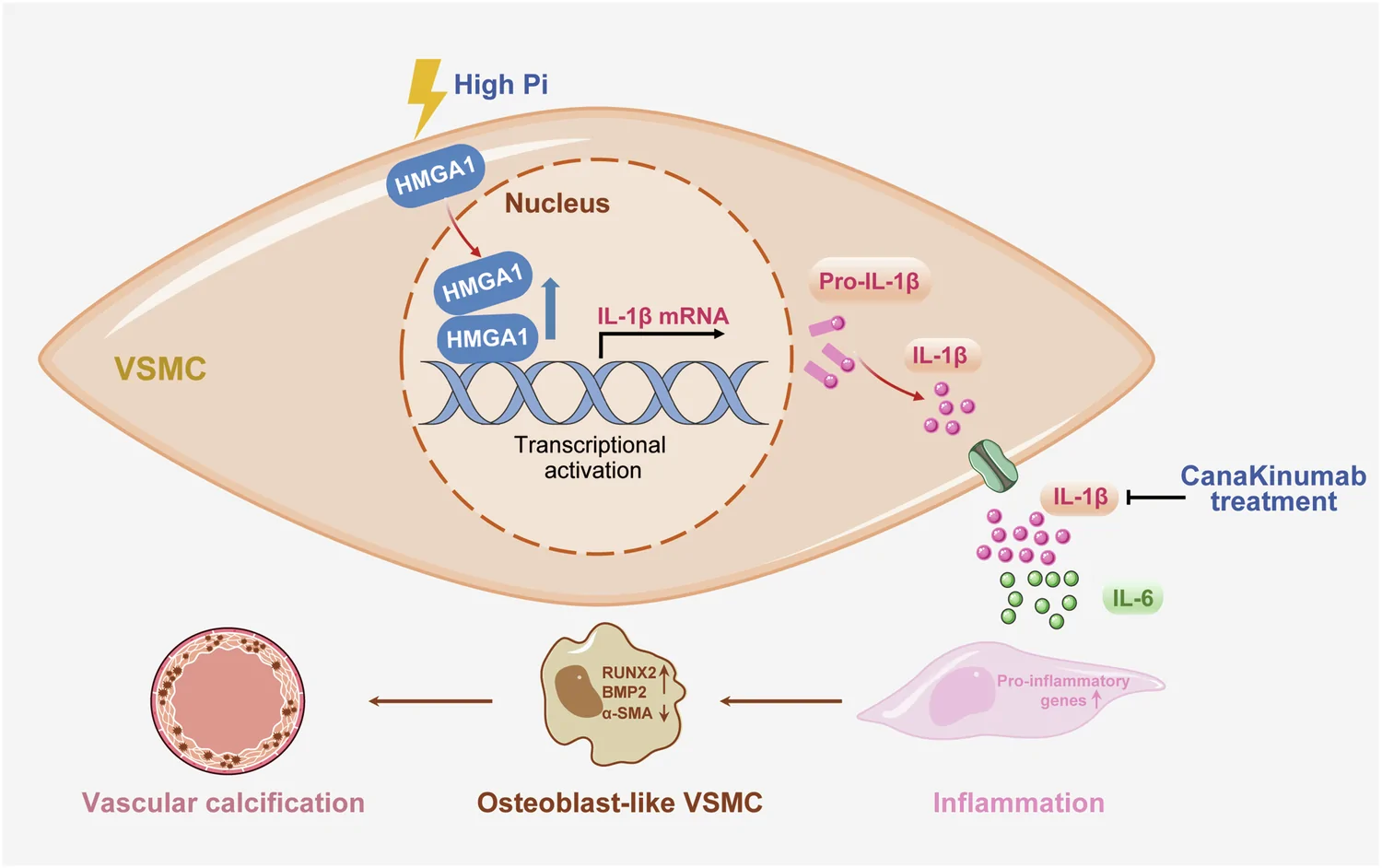
Schematic illustration of HMGA1/IL-1β/IL-6 in osteogenic phenotype transdifferentiation and calcification in the development of chronic kidney disease. High phosphate induces increased HMGA1 expression, triggering direct transcription of IL-1β and thus amplifies the inflammatory signal via IL-6 that ultimately drives VSMC osteogenic transition and calcification.

In summary, our study identifies HMGA1 as a novel transcriptional regulator in VC that promotes the osteogenic differentiation of VSMCs by orchestrating a pro-inflammatory response. Mechanistically, HMGA1 exerts its activation effect on VC by directly binding to the IL-1β promoter and enhancing its transcription, thereby amplifying the inflammatory signal through IL-6 and accelerating the calcification process (Fig. 8). By revealing novel mechanisms underlying the transcriptional regulation of IL-1β, our work provides fundamental new insights into the molecular underpinnings of VC. Consequently, targeted intervention in the HMGA1/IL-1β/IL-6 axis emerges as a promising therapeutic strategy for mitigating this pathological condition.

## Materials and methods

### Data source and analysis

The microarray dataset of high Pi-induced human VSMCs was derived from a published study by Bao et al. [48]. This study identified differentially expressed genes (DEGs) using criteria of *P* < 0.05 and |log2 (fold change)| ≥ 1. Gene Ontology (GO) and Kyoto Encyclopedia of Genes and Genomes (KEGG) analyses were conducted using the Sangerbox online platform (http://www.sangerbox.com). Data visualizations were generated using the Bioinformatics online platform (https://www.bioinformatics.com.cn). Construction of the protein-protein interaction (PPI) network was performed using STRING, and visualized with Cytoscape 3.9.1. Identification of hub genes was achieved through the CytoHubba plugin (confidence >0.7).

### Patients

All procedures involving human samples in this study were performed in compliance with the principles of the Declaration of Helsinki and were approved by the Ethics Committee of the Fourth Hospital of Hebei Medical University (Approval No. 2025KT025). Written informed consent was obtained from all participants prior to their enrollment. A total of 80 patients with CKD receiving maintenance hemodialysis were enrolled from the Fourth Hospital of Hebei Medical University between April and August 2025. Exclusion criteria included individuals under 18 or over 80 years of age, malignancies, acute cardiovascular events, acute infections, and active chronic hepatitis. Relevant biochemical and clinical data were collected from electronic medical records. Peripheral blood samples were collected from patients to analyze serum levels of IL-1β and IL-6 by enzyme-linked immunosorbent assay (ELISA) kits (IL-1β: EIA-0058-Hum; IL-6: EIA-0111-Hum, Elisa Biotech Co., Ltd, Shanghai, China) according to the manufacturer’s instructions. The baseline characteristics of the study participants are presented in Supplementary Table 1.

Radial artery tissue samples (3–5 mm) were obtained from 12 CKD patients during arteriovenous anastomosis surgery prior to dialysis. Following the removal of adipose and adventitial tissues, the specimens were fixed in 4% paraformaldehyde and embedded in paraffin for subsequent analysis.

### Calcification assessment

The presence of VC was evaluated using coronary artery imaging via multidetector computed tomography and quantified by the Agatston score. The Agatston score was independently measured by three analysts using SmartScore software (Definition Flash, Siemens Healthcare, Forchheim, Germany), based on the CT attenuation values of coronary calcified plaques. Stratification was performed according to the coronary calcium score, defining a non-VC group (Agatston score = 0) and a VC group (Agatston score > 0).

### Calcification animal model

All animal experimental procedures were approved by the Institutional Animal Care and Use Committee of the Fourth Hospital of Hebei Medical University and conformed to all relevant regulations on animal research ethics (No. IACUC-4th Hos Hebmu-2024209). Male C57BL/6J mice, aged 8 weeks, were purchased from HuaFuKang Bio Tech (Beijing, China). The animals were housed under specific pathogen-free conditions with a 12-h light/dark cycle and provided with free access to food and water. We randomly assigned the mice to different experimental groups, with 6 mice per group. The control group was fed a standard diet, whereas the CKD-associated VC model group received a special diet containing 0.2% adenine and 1.2% Pi for 12 weeks. The levels of 24-h urine protein and serum creatinine were monitored to assess the establishment of a mouse model of CKD. In detail, we measured the urine protein levels in the supernatant after centrifuging the urine samples using the assay kit (C-035-2-1, Nanjing Jiancheng Bioengineering Institute, Nanjing, China). Total 24-h urinary protein excretion was calculated by multiplying urine volume by urinary protein concentration. Serum was isolated from blood samples, and creatinine levels were determined using a corresponding kit (C011-2, Nanjing Jiancheng Bioengineering Institute, Nanjing, China). Six weeks later, the urine protein and the serum creatinine levels in CKD mice were significantly increased. Then, calcitriol (active vitamin D3, 1 μg/kg, twice a week, Sigma) was subcutaneously administered to the CKD mice to induce media calcification until the end of the experiment.

For VSMC-specific knockdown of HMGA1, recombinant adeno-associated virus serotype 9 (AAV9) carrying HMGA1 shRNA (1 × 10^11^ vg/mL, GeneChem Co., Ltd., Shanghai, China) was administered via tail vein injection. For IL-1β targeted therapy, anti-IL-1β monoclonal antibody (Canakinumab, APExBIO, Shanghai, China) was administered subcutaneously at a dose of 10 mg/kg once every two weeks. Mice were subjected to terminal procedures initiated with 2.5% isoflurane and euthanized by intravenous injection of sodium pentobarbital, followed by peripheral blood collection for the measurement of serum biochemical parameters and ELISA analysis of relevant indicators. Serum levels of Ca (C004-2), Pi (C006-1-1), and alkaline phosphatase (ALP) (A059-2-96) were measured using specific commercial assay kits (Nanjing Jiancheng Bioengineering Institute, Nanjing, China) according to the manufacturer’s instructions. Serum levels of IL-1β and IL-6 were measured by ELISA kits (IL-1β: EIA-0058-Mou; IL-6: EIA-0111-Mou, Elisa Biotech Co., Ltd, Shanghai, China) according to the manufacturer’s instructions. The aortic tissues were excised and randomly divided for subsequent experiments. One half of the samples was stored at −80 °C for molecular detection, while the other half were used for histological analysis.

### Cell culture

VSMCs were isolated from the thoracic aortas of 4-week-old male Sprague-Dawley rats. After aseptically removing the adventitia, the aortic tissues were minced into 1–2 mm² fragments and placed in cell culture flasks. Explant cultures were maintained in Dulbecco’s Modified Eagle Medium (DMEM, 31600-026, Gibco, Waltham, MA, USA) supplemented with 10% fetal bovine serum (FBS, A0500-3010, Cegrogen Biotech, Germany) and incubated at 37 °C under a humidified atmosphere of 5% CO₂. Cells from passages 3 to 5 in good growth conditions were used for subsequent experiments. VSMCs were treated with 10 mM Pi to induce calcification. To evaluate the effect of anti-IL-1β antibody on osteogenic differentiation, cells were incubated with the antibody at concentrations of 1, 5, and 10 μg/mL. Based on the experimental outcomes, a dose of 5 μg/mL was chosen for subsequent studies.

### Plasmids and transfection

Transfection was performed using Lipofectamine 3000 transfection kit (L3000015, Invitrogen, Carlsbad, CA, USA) according to the manufacturer’s instructions. Overexpression of HMGA1 (OE-HMGA1) and IL-1β (OE-IL-1β) was achieved by transfecting VSMCs with pre-designed plasmids. Both the overexpression plasmids and their corresponding negative control plasmids (OE-NC) were purchased from U-Bio Biological Technology Co., Ltd. (Changsha, China). The small hairpin RNA against HMGA1 (sh-HMGA1) was obtained from GeneCopoeia, Inc. (USA). The sequences of shHMGA1 were as follows: shHMGA1-1, AGAATGAGCGAGTCGGTCTCA; shHMGA1-2, CTCCTGACAAGGTTAACTTCC; shHMGA1-3, TGGACAACAGGAAGTAGGATG; sh-NC, GCTTCGCGCCGTAGTCTTA. Following transfection, cells were incubated for 24 h for qRT-PCR analysis and for 48 h for Western blot analysis to evaluate the transfection efficiency.

### Quantitative real-time polymerase chain reaction (qRT-PCR)

Total RNA, extracted from aortic tissues and VSMCs using Trizol Reagent (DP424, TianGen, Beijing, China), was quantified spectrophotometrically for concentration. Reverse transcription of 1 μg total RNA into cDNA was performed in a 20 μL reaction volume with the FastKing RT Kit (KR116, TianGen, Beijing, China), following the manufacturer’s protocol. Quantitative real-time PCR was then conducted using SuperReal PreMixPlus (SYBR Green) (FP205, TianGen, Beijing, China). The relative mRNA expression levels were calculated using the 2^−ΔΔCT^ method, with GAPDH serving as the internal control. All primer sequences are listed in Supplementary Table 2.

### Western blot

Total protein from tissues or VSMCs was extracted using RIPA lysis buffer (WB3100, NCM biotech, Suzhou, China) supplemented with a protease inhibitor cocktail. Protein concentration was determined using a BCA assay kit (KGB2101-500, KeyGEN Biotech, Jiangsu, China). Equal amounts of protein samples were separated by SDS-PAGE and transferred to 0.22 μm polyvinylidene fluoride (PVDF) membranes (03010040001, Sigma-Aldrich, USA). The membranes were blocked with 5% BSA (4240GR100, BioFroxxx, Germany) at room temperature for 1 h, followed by their incubation with primary antibodies at 4 °C overnight. The membranes were incubated with horseradish peroxidase-conjugated secondary antibodies (1:5000 dilution) at room temperature for 1 h. Following washing, the blots were visualized using an enhanced chemiluminescence reagent (P10300, NCM biotech, Suzhou, China) on an Amersham Imager 680 system (ImageQuant 500, Cytiva, USA). The primary antibodies employed in this work are detailed in Supplementary Table 3. Protein expression levels were quantified by analyzing the band intensity with ImageJ software, and normalized to corresponding internal controls. See original western blots in Supplementary Fig. 4.

### Immunofluorescence staining

VSMCs were fixed with 4% paraformaldehyde at room temperature for 30 min, followed by being permeabilized with 0.3% Triton X-100 for 15 min to enhance membrane permeability. Cells were blocked with 5% goat serum at 37 °C for 1 h to prevent nonspecific binding. After removing the blocking solution, the cells were incubated with primary antibodies at 4 °C overnight in a humidified chamber. The next day, the cells were incubated with fluorophore-conjugated secondary antibodies for 1 h at room temperature while being protected from light. Nuclei were counterstained with DAPI (S2110, Solarbio, Beijing, China) for 10 min at room temperature in the dark. All incubation steps were followed by extensive washing with PBS.

For mouse aortic tissue sections, deparaffinization with xylene and graded alcohols was performed first, followed by antigen retrieval using EDTA buffer (C1034, Solarbio, Beijing, China). Subsequent staining steps were consistent with the cell experimental protocol described above. Finally, images were observed and captured using a fluorescence microscope (Axioscope5, ZEISS, Germany). Detailed information regarding the antibodies used in this study is provided in Supplementary Table 3. Fluorescence intensity was quantified using ImageJ software. After background subtraction, the mean fluorescence intensity values were normalized to the mean of the control group and presented as fold change relative to the control.

### Immunohistochemical (IHC) staining

Paraffin-embedded tissue sections were dewaxed in xylene and rehydrated through a graded ethanol series. After antigen retrieval using a high-pressure heating method with EDTA buffer, the endogenous peroxidase activity was quenched by incubation with 3% hydrogen peroxide (H₂O₂) for 10 min at room temperature. Following washing with PBS, the sections were blocked with 5% normal goat serum for 1 h at room temperature to minimize nonspecific binding. The sections were then incubated with primary antibodies diluted in antibody diluent overnight at 4 °C in a humidified chamber. After washing three times with PBS, the sections were incubated with secondary antibodies for 40 min at room temperature. Following another three washes with PBS, the 3,3’-diaminobenzidine (DAB) (ZLI-9018, ZSGB-BIO, Beijing, China) staining working solution was prepared according to the manufacturer’s instructions. Following color development, the slides were immersed in distilled water to terminate the reaction. The sections were counterstained with hematoxylin (KeyGen, China), differentiated in acid alcohol, washed three times with tap water, and subsequently mounted for microscopic examination. Antibody details are listed in Supplementary Table 3. IHC staining was quantified using ImageJ software by measuring the average optical density, and the values were expressed as fold change relative to the control group.

### Alizarin red staining

Alizarin red staining was employed to identify calcification in VSMCs. After removing the culture medium, the cells were washed three times with PBS and fixed with 4% paraformaldehyde for 30 min. Following another three washes with PBS, the cells were stained with 1% Alizarin Red S solution (pH 4.2, G1452, Solarbio, Beijing, China) at 37 °C for 15 min. Positive staining, indicating calcium deposits, was observed as orange-red areas.

### Von Kossa staining

Von Kossa staining was performed to assess calcification in the aorta. Briefly, 5 μm paraffin sections of arterial tissues were deparaffinized and rehydrated. The sections were then incubated in 5% silver nitrate (G3282, Solarbio, Beijing, China) solution under ultraviolet light for 1 h, followed by three washes with distilled water. Subsequently, the sections were treated with 5% sodium thiosulfate for 1 min. After another wash with distilled water, the sections were counterstained with eosin for 15 s. Finally, the sections were washed, mounted with neutral mounting medium, and imaged under a light microscope. Calcified areas appeared as dark brown deposits.

### Quantification of calcium content

By using a commercial calcium assay kit (S1063, Beyotime, Shanghai, China), the calcium content in supernatants from VSMC lysates and aortic tissue homogenates was determined. The protein concentration in the supernatant was measured using a BCA protein assay kit. The final calcium content was normalized to the protein concentration and expressed as mg/g protein.

### Cell viability analysis

Cell viability was measured using the Cell Counting Kit-8 (CCK-8) reagent (C6005, NCM Biotech, Suzhou, China) in accordance with the manufacturer’s instructions. Briefly, VSMCs were seeded in 96-well plates at a density of 5 × 10^3^ cells per well in 100 μL of complete medium and pre-cultured for 24 h. The cells were then treated with various concentrations of canakinumab for the indicated durations. After treatment, the drug-containing medium was aspirated, and the cells were incubated with 100 μL of fresh medium containing 10 μL of CCK-8 solution for 1 h at 37 °C. The absorbance was measured at a wavelength of 450 nm using a microplate reader. Wells containing only culture medium and CCK-8 solution served as the blank control.

### Chromatin immunoprecipitation (CHIP)

ChIP assay was conducted according to the protocol of the EpiQuik ChIP Kit (P-2002, Epicentre, NY, USA). Cells were cross-linked with 1% formaldehyde for 10 min at room temperature, and the reaction was quenched with 0.125 mol/L glycine. The cells were then lysed, and the chromatin was fragmented into pieces of 200–1000 bp using a sonicator (Model KBS-150, Kunsan Ultrasonic Instrument Co., Jiangsu, China). An aliquot (5 μL) of the sonicated chromatin was saved as the input control. The remaining chromatin supernatant was incubated with an anti-HMGA1 antibody or normal rabbit IgG at room temperature for 90 min. Subsequently, Protein A/G-coated magnetic beads were added to capture the antibody-chromatin complexes. After extensive washing, the immunoprecipitated complexes were eluted from the beads. The cross-links were reversed, and the DNA was digested with Proteinase K and purified. The sequences of primers are listed in Supplementary Table 4.

### Dual-luciferase reporter assay

To validate the transcriptional regulation of the target gene by HMGA1, a dual luciferase reporter assay was performed. We constructed wild-type (WT) and mutant (Mut) plasmids containing the predicted binding sites. HEK293T cells seeded in 96-well plates were co-transfected using Lipofectamine 3000 with the WT or Mut promoter reporter plasmid, along with either an HMGA1-overexpression plasmid or an empty vector control. After 48 h of transfection, Firefly and Renilla luciferase activities were measured sequentially using the dual-luciferase reporter assay (E2940, Promega, Madison, WI, USA). The Firefly luciferase activity was normalized to the Renilla luciferase activity in each well.

### Statistical analysis

For animal models, random allocation was performed using a random number table, and experiments involving mice were conducted in a blinded manner. The investigator was blinded to group allocation during the experiments. For cell experiments, VSMCs were randomly assigned to each experimental condition, and samples were analyzed in a blinded fashion. Data are presented as the mean ± standard deviation (SD) from at least three independent biological replicates per group. All statistical analyses were conducted with SPSS 27.0 software and GraphPad Prism 10 software. The normality of the data distribution was verified using the Shapiro–Wilk test. Statistical comparisons between two groups were performed using an unpaired two-tailed Student’s *t* test. One-way analysis of variance (ANOVA) was applied for multiple-group comparisons, followed by Tukey’s post-hoc pairwise comparisons. The associations between two variables were assessed by Spearman’s correlation analysis. A *P*-value of less than 0.05 was considered statistically significant. In each set of experiments, the sample size was chosen to ensure adequate power of detection.

## Supplementary information

supplementary figure4

supplemental material

supplementary figure1

supplementary figure2

supplementary figure3

## Data Availability

The original data for western blots, as well as the prime sequence of genes are available in the supplementary results. All source data underlying the graphs presented in the figures were available in Supplementary Data.
