# Supplementary material for: HMGA1 accelerates vascular smooth muscle cell osteogenic phenotype transdifferentiation by elevating pro-inflammatory cytokine IL-1β transcription in chronic kidney disease
Source: Cell Death Discov. 2026 Aug 4;12:435. doi: 10.1038/s41420-026-03271-z (PMC13720042; doi:10.1038/s41420-026-03271-z)
Supplement: Supplementary file 1 [file 41420_2026_3271_MOESM2_ESM.docx]

**H****MGA1 accelerates** **vascular smooth muscle cell** **osteogenic phenotype transdifferentiation by elevating pro-inflammatory cytokine IL-1β transcription in chronic kidney disease**

**Binhong Yang^1, 2, 3^, Meijuan Cheng^1, 2, 3^, Jingjing jin^1, 2, 3^****, Kaixing Yan^1, 2, 3^, Haoqi Sun^1, 2, 3^, Xinyue Yang^1, 2, 3^, Yunhui Wu^1, 2, 3^, Qi An^1, 2, 3^, Dongxue Zhang^1, 2, 3^, Lixin Chang^1, 2, 3^, Shenglei Zhang^1, 2, 3^, Yaling Bai^1, 2, 3^ and Jinsheng Xu^1, 2, 3*^.**

1Department of Nephrology, The Fourth Hospital of Hebei Medical University, Shijiazhuang P.R. China

2Hebei Clinical Research Center for Chronic Kidney Disease

3Hebei Key Laboratory of Vascular Calcification in Kidney Disease

* To whom correspondence should be addressed:

Xu Jinsheng, MD, PhD

E-mail: 46400262@hebmu.edu.cn

**Figure Legends**

**Supplementary Fig. 1** **IL-1β and IL-6 were upregulated in the CKD-VC mouse model and in Pi-stimulated VSMCs.** (A) Representative Von Kossa and immunohistochemical staining of IL-1β and IL-6 in control and CKD vessels (n=3). Scale bars, 200 μm. (B) Alizarin red staining of VSMCs cultured with Pi for 7 days (n=3). (C) The mRNA expression levels of IL-1β and IL-6 in VSMCs were analyzed by qRT-PCR after 7 days of Pi induction (n=3). Data are presented as mean±SD. * *P* < 0.05, ** *P* < 0.01, *** *P* < 0.001. CKD chronic kidney disease, VC vascular calcification, Pi phosphate, VSMCs vascular smooth muscle cells.

**Supplementary Fig. 2 The pro-inflammatory** **IL-1β overexpression aggravated VSMC osteogenic differentiation and calcification.** (A) qRT-PCR showed the transfection efficiency of IL-1β overexpression (n=3). (B) The protein levels of pro-IL-1β, IL-1β, and IL-6 in VSMCs with IL-1β overexpression (n=3). (C) Calcium content in VSMCs from different experimental groups (n=3). (D) Alizarin red staining in VSMCs pre-transfected with the indicated treatment (n=3). (E) Protein levels and quantitative analysis of RUNX2, BMP2, and α-SMA in VSMCs with IL-1β overexpression (n=3). Data are presented as mean±SD. * *P* < 0.05, ** *P* < 0.01, *** *P* < 0.001. VSMCs vascular smooth muscle cells.

**Supplementary Fig. 3 Characterization of adenine diet-induced CKD mice with HMGA1 deletion.** (A) Body weight (n=6) (B) Macroscopic observation of the kidney (C) 24-hour urinary protein (n=6) (D) Serum creatinine (n=6) in the indicated groups. Data are presented as mean±SD. * *P* < 0.05, ** *P* < 0.01, *** *P* < 0.001. CKD chronic kidney disease.

**Supplementary Fig. 4 Original western blot.**

**Supplementary Table 1.** Comparison of baseline characteristics between CKD patients with and without VC.

| Characteristics | Non-VC (n=31) | VC (n=49) | *P* value |
| --- | --- | --- | --- |
| Age (years) | 46.06±16.31 | 59.06±13.19 | <0.001 |
| Male (%) | 16 (51.6%) | 32 (65.3%) | 0.223 |
| BMI (kg/m²) | 22.08±5.82 | 22.05±3.60 | 0.979 |
| Duration of dialysis (years) | 5.51 (3.37-9.43) | 5.27 (2.51-12.96) | 0.953 |
| Ca (mmol/L) | 2.18±0.20 | 2.13±0.19 | 0.200 |
| Pi (mmol/L) | 1.62 (1.39-2.03) | 1.54 (1.30-1.78) | 0.345 |
| K (mmol/L) | 4.80 (4.50-5.40) | 4.80 (4.60-5.20) | 0.843 |
| Na (mmol/L) | 137.52±3.35 | 137.43±3.01 | 0.904 |
| Hemoglobin (g/L) | 115.00 (110.00-121.00) | 118.00 (112.00-123.00) | 0.262 |
| Ferritin (ug/L) | 484.30 (197.00-719.90) | 304.80 (162.70-461.65) | 0.070 |
| ALB (g/L) | 40.98±2.72 | 39.88±2.42 | 0.062 |
| ALP (g/L) | 87.90 (81.10-113.10) | 99.60 (72.60-119.20) | 0.672 |
| iPTH (pg/ml) | 196.60 (102.30-299.90) | 170.00 (95.40-279.85) | 0.502 |
| 25-OH VD (ng/ml) | 13.82 (10.51-22.13) | 14.37 (10.10-20.70) | 0.778 |
| CRP (mg/L) | 2.88 (1.99-5.57) | 2.83 (2.02-5.22) | 0.794 |
| Pre-dialysis β2-microglobulin | 29.45 (26.76-32.61) | 28.70 (24.64-32.08) | 0.194 |
| Post-dialysis β2-microglobulin | 11.10 (8.89-14.24) | 11.55 (10.14-13.63) | 0.671 |

CKD: chronic kidney disease; VC: vascular calcification; BMI: body mass index; Ca: calcium; Pi: phosphate; K: potassium; Na: natrium; ALB: albumin; ALP: alkaline phosphatase; iPTH: intact parathyroid hormone; 25-OH VD: 25-Hydroxy Vitamin D; CRP: C-reaction protein. Continuous variables are presented as mean ± SD or median (25-75% quartiles), and categorical variables as n (%). Statistical significance was assessed using Student’s t test (all characteristics except sex) or nonparametric Mann Whitney U test (sex).

**Supplementary Table 2.** Primers used for RT-qPCR.

| Gene | Forward primer | Reverse primer |
| --- | --- | --- |
| mus-HMGA1 | AGTGCCAACTCCGAAGAGACCT | TTGGTTTTCCTCCCTGGAGCTGT |
| mus-IL-1β | AACTCAACTGTGAAATGCCACC | CATCAGGACAGCCCAGGTC |
| mus-IL-6 | GCCTTCTTGGGACTGATGCT | GACAGGTCTGTTGGGAGTG |
| mus-GAPDH | AGGTCGGTGTGAACGGATTTG | GGGGTCGTTGATGGCAACA |
| rat-HMGA1 | AGTCAGAAGGAGCCCAGTGA | CTCCCCGGAGTTGTGGTAAC |
| rat-HMGA2 | CACATCAGCCCAGGGACAAC | TGGGTCTCTTAGGAGAGGGC |
| rat-MMP1 | GCTTCTTTGGCTTCCCTAGC | CCTGGATCCATGGACTGTGT |
| rat-IL-1β | GGGATGATGACGACCTGCTA | TGTCGTTGCTTGTCTCTCCT |
| rat-IL-6 | CTCTGGTCTTCTGGAGTTCCG | GGAAGTTGGGGTAGGAAGGAC |
| rat-GAPDH | CAAGGCTGAGAATGGGAAGC | GAAGACGCCAGTAGACTCCA |

**Supplementary Table 3.** Antibodies used this study.

| Antibody | Manufacturer | Dilution ratio |
| --- | --- | --- |
| Anti-HMGA1 | Santa Cruz, sc-393213 | WB: 1:1000; IF/IHC: 1:200  ChIP: 4μg |
| Anti-IL-1β | Proteintech, 26048-1-AP | WB: 1:1000; IF: 1:50;  IHC: 1:100 |
| Anti-IL-6 | Proteintech, 26404-1-AP | WB: 1:1000; IF: 1:50;  IHC: 1:100 |
| Anti-BMP2 | Proteintech, 66383-1-lg | WB: 1:1000; IHC: 1:100 |
| Anti-RUNX2 | Abcam, ab76956 | WB: 1:1000; IHC: 1:200 |
| Anti-α-SMA | Proteintech, 67735-1-lg | WB: 1:1000; IF: 1:200 |
| Anti-GAPDH | Proteintech, 60004-1-lg | WB: 1:1000 |
| Anti-rabbit | Report, S1002 | WB: 1:5000 |
| Anti-mouse | Report, S1001 | WB: 1:5000 |
| Goat Anti-mouse IgG | Abbkine, A23410 | IF: 1:100 |
| Goat Anti-rabbit IgG | Abbkine, A23220 | IF: 1:200 |
| Rabbit control IgG | ABclonal, AC005 | ChIP: 1μl |

**Supplementary Table 4.** Primers used for ChIP-PCR.

| Gene | Forward primer | Reverse primer |
| --- | --- | --- |
| IL-1β-1 | GTTTTTCTGTTTGGGGTTC | CCATTGGATAACACAGACATT |
| IL-1β-2 | TCTTGGGTTGCTTGATACTGC | AGAACGAGCCTGGATTGAGT |
| IL-1β-3 | TGTTCCAGACATCAGGACTTC | TGTGGTGGAAACAGACATTAT |

**Supplementary** **Table 5.** OR of VC for IL-1β and IL-6 estimated by logistic regression.

| Characteristics | Model 1 OR (95% CI) | *P-*value | Model 2 OR (95% CI) | *P-*value |
| --- | --- | --- | --- | --- |
| Serum IL-1β | 1.061 (1.019-1.105) | 0.004 | 1.059 (1.014-1.107) | 0.010 |
| Serum IL-6 | 2.466 (1.540-3.950) | <0.001 | 2.992 (1.669-5.363) | <0.001 |

Model 1: unadjusted; Model 2: adjusted for age. OR: odds ratios.

**Supplementary Table 6.** The top 15 candidate HMGA1 target genes ranked by correlation coefficient of expression.

| Gene symbol | r | \|log2FC\| | *P* value |
| --- | --- | --- | --- |
| IL-6 | 0.99 | 1.05 | <0.005 |
| REEP1 | -0.98 | 1.39 | <0.005 |
| TLR3 | 0.98 | 1.53 | <0.005 |
| SLC5A1 | -0.96 | 1.38 | <0.005 |
| ADAMTSL1 | 0.96 | 1.12 | <0.005 |
| C3 | 0.96 | 1.20 | <0.005 |
| RNASEL | 0.96 | 1.08 | <0.005 |
| CASP12 | 0.96 | 2.00 | <0.005 |
| CXCL6 | 0.94 | 1.80 | <0.005 |
| NTRK3 | 0.94 | 1.46 | <0.005 |
| MMP1 | 0.94 | 2.88 | <0.005 |
| IL-1β | 0.93 | 2.69 | <0.005 |
| F2RL1 | 0.93 | 1.13 | <0.005 |
| PLAU | 0.93 | 1.01 | <0.005 |
| TNFAIP3 | 0.93 | 1.61 | <0.005 |
